# Lightweight Vehicle Detection Based on Mamba_ViT

**DOI:** 10.3390/s24227138

**Published:** 2024-11-06

**Authors:** Ze Song, Yuhai Wang, Shuobo Xu, Peng Wang, Lele Liu

**Affiliations:** School of Information and Electrical Engineering, Shandong Jiaotong University, Jinan 250357, China; 18764459319@163.com (Z.S.);

**Keywords:** artificial intelligence, deep learning, object detection, vehicle detection, lightweight

## Abstract

Vehicle detection algorithms are essential for intelligent traffic management and autonomous driving systems. Current vehicle detection algorithms largely rely on deep learning techniques, enabling the automatic extraction of vehicle image features through convolutional neural networks (CNNs). However, in real traffic scenarios, relying only on a single feature extraction unit makes it difficult to fully understand the vehicle information in the traffic scenario, thus affecting the vehicle detection effect. To address this issue, we propose a lightweight vehicle detection algorithm based on Mamba_ViT. First, we introduce a new feature extraction architecture (Mamba_ViT) that separates shallow and deep features and processes them independently to obtain a more complete contextual representation, ensuring comprehensive and accurate feature extraction. Additionally, a multi-scale feature fusion mechanism is employed to enhance the integration of shallow and deep features, leading to the development of a vehicle detection algorithm named Mamba_ViT_YOLO. The experimental results on the UA-DETRAC dataset show that our proposed algorithm improves mAP@50 by 3.2% compared to the latest YOLOv8 algorithm, while using only 60% of the model parameters.

## 1. Introduction

As the number of vehicles continues to grow, cars have become an indispensable part of our lives. However, the transportation system remains inefficient, resulting in longer waiting times at intersections, increased environmental pollution, higher accident rates, and worsening traffic congestion. Therefore, intelligent vehicle detection has become a key component in the development of intelligent traffic management systems [1,2]. This technology aids in reducing traffic accidents and mitigating congestion [3]. Nonetheless, in real-world scenarios, vehicle detection algorithms need to address challenges such as varying environments, variability in vehicle shapes, occlusion, and the computational complexity of the algorithm. Thus, researching an efficient and lightweight vehicle detection algorithm is of considerable academic importance.

Currently, extensive research has been conducted on vehicle detection algorithms [4,5]. These efforts have focused on CNN-based methods (convolutional neural networks). CNN-based methods can be subdivided into one-stage methods and two-stage methods. One-stage methods include YOLOv4, YOLOv7, YOLOv8, and SSD [6,7,8]. These methods directly generate vehicle detection results through a single neural network, simplifying vehicle detection into regression and classification tasks. Two-stage methods include R-CNN, Fast R-CNN, Faster R-CNN, and Mask R-CNN [9,10,11,12]. Two-stage methods first use search algorithms to select candidate regions, followed by the application of CNN models to extract features and classify these candidate regions. Compared to one-stage vehicle detection methods, two-stage methods offer higher detection accuracy. However, they also involve higher computational costs and a larger number of model parameters. Consequently, in practical vehicle detection applications, most researchers prefer one-stage methods. For example, Wang et al. [13] developed an enhanced k-median clustering algorithm based on YOLOv3. By implementing parallel branches on the backbone network, they mitigated model instability caused by outliers and enhanced weak features, which is particularly useful for small-scale target detection. Kasper-Eulaers et al. [14] utilized YOLOv5 for truck detection and successfully identified heavy trucks in winter rest areas, allowing for real-time prediction of parking space occupancy. However, the model had difficulty detecting heavy trucks obscured by other vehicles, resulting in a high missed detection rate. Xudong Dong et al. [15] introduced an optimized lightweight YOLOv5 vehicle detection method. By incorporating C3_Ghost and Ghost modules, a Convolutional Block Attention Module (CBAM), and CIoU_Loss, this method substantially improved vehicle detection accuracy while reducing the number of model parameters. Zhang Xiliu et al. [16] introduced an enhanced vehicle detection model based on the YOLOX network, incorporating multi-scale feature fusion. This model introduced a Ghost-CSP structure based on depthwise separable convolution, replaced the max-pooling method with Softpool, integrated a coordinate attention mechanism, and employed Focal Loss as the confidence loss function. These enhancements were designed to enhance vehicle detection performance under real-world conditions.

Although the aforementioned methods are significant for vehicle detection, certain limitations remain. Firstly, while most methods enhance vehicle detection accuracy under certain conditions, they primarily rely on CNNs for feature extraction [17]. CNNs possess strong local modeling capabilities [18,19], which are effective for capturing local details. However, in real traffic scenarios, relying solely on local features is insufficient for fully comprehending vehicle image data. Secondly, some researchers have incorporated Transformers into the backbone network to enhance the model’s global modeling capabilities [20,21]. While this approach improves global feature extraction, the quadratic computational complexity of Transformers significantly raises the computational demands of the algorithm. Lastly, the process of aggregating features from shallow to deep layers in these algorithms relies on CNNs. Shallow features typically capture local details such as edges and textures, which CNNs can effectively extract. In contrast, deep features represent more complex aspects like contours, shapes, or specific components. Relying solely on CNNs to capture deep features can lead to a loss of global information and can thus negatively affect vehicle detection performance.

To address these issues, we propose a lightweight vehicle detection algorithm based on Mamba_ViT. The main contributions of this paper are as follows:

- We propose an efficient feature extraction network named Mamba_ViT. This network comprises two modules: Mamba_F and iRMB_F. These modules are designed to separate shallow features from deep features and process them independently through different network structures. The iRMB_F module focuses on extracting shallow features, such as edges and textures, while the Mamba_F module is responsible for capturing deep features, such as object contours and shapes. This separation approach optimizes the feature extraction process and reduces the loss of vehicle information.
- In the Mamba_ViT network, we incorporate a multi-scale feature fusion mechanism. By integrating features from different scales, this approach facilitates more efficient fusion of both shallow and deep features captured by Mamba_ViT.
- On the UA-DETRAC dataset, our proposed algorithm achieves a 3.2% improvement in mAP@50 compared to the latest YOLOv8 algorithm, while utilizing only 60% of the parameters of YOLOv8.

## 2. Related Work

### Vehicle Detection

Vehicle detection is used to identify vehicles within a designated area and precisely classify vehicle types to accurately determine their positions [22]. Object detection has been a central focus in the field of computer vision. However, owing to the diversity of vehicle appearances and the presence of varying dynamic states during detection, vehicle detection remains one of the most fundamental and challenging tasks in the domain of object detection [23].

In the early stages of research, vehicle detection predominantly relied on traditional methods. These traditional vehicle detection methods typically relied on manual extraction of vehicle features from video sequences, followed by classification and recognition of the extracted features. The most notable manual feature extraction methods include Histogram of Oriented Gradients (HOG) [24], Haar features [25], and Scale-Invariant Feature Transform (SIFT) [26]. Amit et al. [27] proposed a novel strong classifier based on machine learning. This method forms better decision boundaries by utilizing more features while leveraging fewer features to exclude many negative samples. The approach involves training generative weak classifiers with HOG features and discriminative weak classifiers with Haar features. Jheng et al. [28] proposed a symmetry-based forward vehicle detection and collision warning system, which utilizes the symmetric shadow features of vehicles for recognition and can operate on smartphones. Munajat et al. [29] introduced an innovative vehicle detection method that employs corner detection and line adjacency graph features. This method creates a binary image through a thresholding process and detects the corner points of each object in each frame to achieve vehicle detection and tracking. R. K. Satzoda et al. [30] proposed a vehicle detection method based on the symmetry of the vertical centerline of the rear of vehicles. They achieved effective vehicle detection by locating the regions of interest (ROI) that contain vehicles in images with high levels of symmetry. However, handcrafted feature extraction methods rely on prior knowledge. In real-world scenarios, there are many objective challenges such as occlusion and deformation. Consequently, traditional vehicle detection algorithms often struggle to meet the accuracy and robustness demands in practical applications [31,32,33].

With the continuous development of deep learning, feature extraction methods for vehicle images have evolved significantly. Unlike traditional vehicle detection methods, deep learning-based approaches do not require manual feature selection; they can autonomously extract features and learn from them [34,35]. Deep learning-based vehicle detection algorithms are generally categorized into two approaches: two-stage object detection frameworks and one-stage object detection frameworks. The two-stage approach involves two main tasks: first, generating vehicle region proposals, and second, performing vehicle object detection. This method initially generates vehicle region proposal boxes indicating areas where vehicles are likely to be present and then uses a prediction network to detect vehicles within these proposal boxes [36]. In contrast, the single-stage approach eliminates the generation of vehicle proposal boxes by integrating vehicle recognition and detection within a single network, or by setting a series of anchor points on feature maps to directly predict the vehicle’s center and bounding box. In contrast to the two-stage algorithm, the generation of suggested regions is eliminated and the single-stage approach directly predicts the target region on the feature map. Therefore, the computational cost is significantly lower than that of the two-stage algorithms. The most notable example of a one-stage approach is the YOLO (You Only Look Once) series of algorithms.

YOLO is the first one-stage object detector. It performs detection by dividing the entire image into multiple regions and applying a unified neural network across the entire image. The model then predicts the bounding boxes and probabilities for each region, enabling rapid detection. While YOLOv1 achieves relatively fast detection speeds, its performance declines when detecting targets that are close together or smaller in size. Building on YOLOv1, Joseph et al. made a series of improvements, introducing YOLO9000 [37] and YOLOv3 [38]. They enhanced the loss function by integrating structures like the Feature Pyramid Network (FPN). In 2020, Alexey Bochkovskiy et al. introduced YOLOv4, which uses CSPDarknet53 as the backbone network and incorporates data augmentation techniques and an improved loss function, achieving high accuracy with reduced model parameters. In the same year, Jocher Glenn introduced YOLOv5. YOLOv5, similar to YOLOv4, also uses CSPDarknet53 as the backbone network but employs a combination of the Feature Pyramid Network (FPN) and the Pixel Aggregation Network (PAN) in the network’s neck. Subsequently, X. Ding et al. introduced YOLOv7, which features a new Efficient Layer Aggregation Network (E-ELAN) to further enhance detection accuracy. In January 2023, Ultralytics released YOLOv8, representing a major advancement in the YOLO series. YOLOv8 introduces comprehensive enhancements and multifunctionality, further improving its performance and flexibility in real-time object detection and related computer vision tasks. However, the backbone network of the YOLO algorithm relies on convolutional neural networks. Although convolutional neural networks have strong local modeling capabilities, it is difficult to perfectly interpret vehicle image data in real-world scenarios by relying on local features. Some researchers have chosen to address this issue [20,21] by adding the Transformer to the feature extraction network to enhance the algorithm’s ability to sense global information. However, the quadratic computational complexity of the Transformer significantly increases the algorithm’s computational cost. Furthermore, the gap between shallow features and deep features is so large that relying solely on convolutional or Transformer aggregated features can lead to incomplete information. Therefore, this paper proposes a lightweight vehicle detection method based on Mamba_ViT.

## 3. Method

### 3.1. Overall Architecture

**Figure 1:**
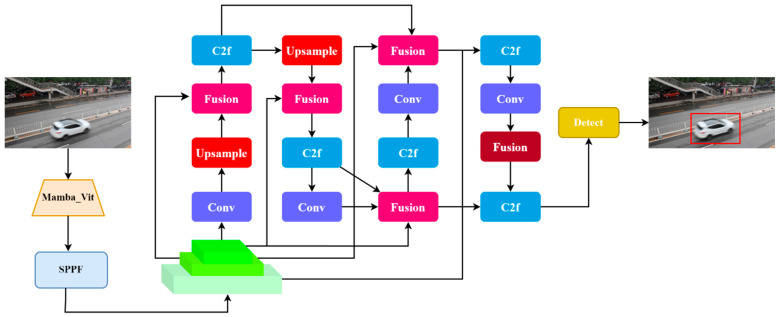
Mamba_ViT_YOLO.

This paper proposes a lightweight vehicle detection algorithm based on Mamba_ViT_YOLO, as illustrated in Figure 1. First, we introduce an efficient deep learning model named Mamba_ViT, which combines the advantages of Mamba and Vision Transformer (ViT) to effectively interpret vehicle image data while maintaining low computational costs. Subsequently, we use the Mamba_ViT model as the feature extraction network to develop a vehicle detection algorithm suitable for real-world scenarios. This algorithm not only achieves superior performance but also significantly reduces computational requirements, making it suitable for various practical applications.

### 3.2. Mamba_ViT

**Figure 2:**
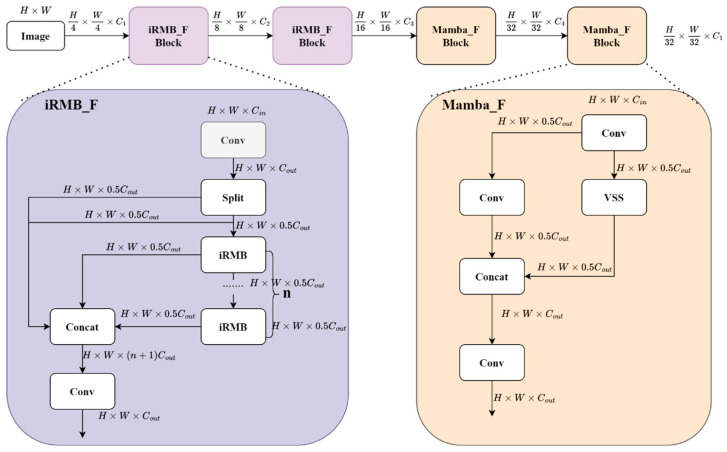
Mamba_ViT.

In previous research work, most researchers [39,40,41,42] have used a CNN as the sole feature extraction unit to construct the network architecture. Although this method can effectively extract features, in real-world traffic scenarios, the presence of complex factors prevents the model from fully interpreting vehicle information, thus negatively impacting vehicle detection performance. Therefore, we propose a hybrid feature extraction architecture, Mamba_ViT, as shown in Figure 2. In the design process, we adopt a standard four-stage design approach [43,44,45]. Each stage consists of a series of building blocks and performs a downsampling operation before each stage. The input resolution for each stage progressively shifts from a stride of 4 to a stride of 32.

**Figure 3:**
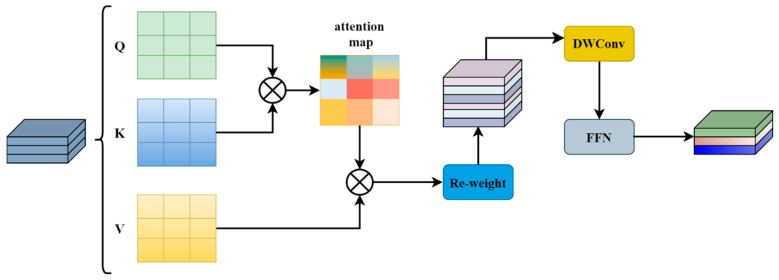
iRMB.

In order to better understand vehicle features, we use different aggregation strategies for shallow features and high-level features. Shallow features usually contain local details such as edges and textures. At the initial stage, we designed the iRMB_F module, featuring local sensing capabilities, shown in Figure 2. iRMB_F uses Inverted Residual Mobile Block (iRMB) [46] as the main building operator. The iRMB is shown in Figure 3. The iRMB has similarities with the inverse inverted residual structure (IRB) in traditional convolutional neural networks (CNNs), where an expansion layer expands the image channels into a high-dimensional space and aggregates features within that space using an efficient operator, and then a contraction layer reduces the channel dimensions. While the IRB uses depth-separable convolution as its efficient operator, the iRMB uses an improved combination of windowed attention and depth-separable convolution. As a result, the iRMB’s receptive field is localized, making it effective in capturing fine-grained information in images. Moreover, the combination of a self-attention mechanism and depth-separable convolution allows the features within each window to interact and fuse with each other, which enhances the representation of local features and improves the model’s capacity to capture shallow local details. In addition to this, windowed attention and depth-separable convolution can effectively reduce computational complexity and parameter count. The iRMB can be represented as follows:

(1) $$\begin{array}{c} X_{i}=Expansion\left(X\right)\left(\epsilon R^{\lambda C\times H\times W}\right) \end{array}$$

(2) $$\begin{array}{c} X_{mid}=\left(DW_{Conv},Skip\right)\left(EW\_MHSA(X_{i})\right)\left(\epsilon R^{\lambda C\times H\times W}\right) \end{array}$$

(3) $$\begin{array}{c} X_{out}=Shrink\left(X_{mid}\right)\left(\epsilon R^{C\times H\times W}\right) \end{array}$$

where λ is the channel scaling factor, $DW_{Conv}$ is depthwise separable convolution, Skip is the skip connection, and EW_MHSA is the improved window attention mechanism.

**Figure 4:**
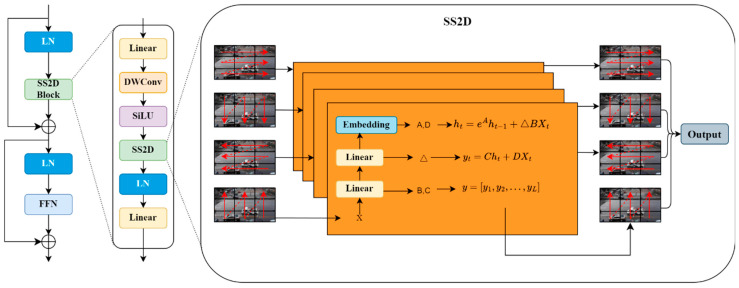
VMamba.

Deep features can represent the contours, shapes, or specific parts of an entire object, and are more abstract than the low-level features (e.g., edges, textures). For such features, local features captured by the CNN or iRMB alone cannot be fully interpreted. Therefore, at deeper stages, we designed the Mamba_F module with integrated perception capability. This module uses VMamba as a core operator. The core of VMamba [47] is a collection of visual state space (VSS) blocks with 2D selective scanning (SS2D) modules. The core of the VSS blocks with 2D selective scanning (SS2D) modules is shown in Figure 4. SS2D scans the image by dividing it into multiple sub-regions, with four scanning routes inside each sub-region via a selective scanning mechanism. This approach not only captures local features, but also effectively aggregates global information and enhances the representation of complex patterns. Subsequently, by employing complementary one-dimensional traversal paths, each pixel in the image can efficiently aggregate information from all other pixels in different directions. The SS2D working principle is shown in Figure 4. Overall, the Mamba_F module effectively achieves an integrated local and global perception of deep features by synergizing SS2D and the VSS blocks. This integrated perception capability enables the model to understand and detect vehicle targets more perfectly when processing high-level abstract features. Moreover, the VSS blocks have linear complexity and do not increase the computational cost of the model.

### 3.3. IRMB_F

The iRMB_F follows the design principles of the C2f module. Specifically, first, a 1 × 1 convolutional layer is used to linearly combine and mix the input feature map channels, thereby reorganizing and merging the features. Then, to reduce computational redundancy, only a portion of the channels is selected for subsequent operations. The selected channels are processed using the iRMB module, repeated *n* times. Finally, the features processed through each layer of the iRMB module are concatenated with the unselected channel features, and a 1 × 1 convolutional layer is used to integrate the channel information and merge different feature representations. The iRMB_F can be represented as follows:

(4) $$\begin{array}{c} X_{f}=Conv\left(X\right)\left(\in R^{C\times H\times W}\right) \end{array}$$

(5) $$\begin{array}{c} X_{s},X_{ns}=Split\left(X_{f}\right) \end{array}$$

(6) $$\begin{array}{c} X_{j}=iRMB\left(X_{s}\right)\left(\in R^{\alpha C\times H\times W}\right) \end{array}$$

(7) $$\begin{array}{c} X_{j+1}=iRMB\left(X_{j}\right)\;1\leq j\leq n \end{array}$$

(8) $$\begin{array}{c} X_{cat}=Concat\left[X_{ns},\left(X_{1},X_{2},X_{3},\ldots ,X_{n}\right)\right]\left(\in R^{(n+1)C\times H\times W}\right) \end{array}$$

(9) $$\begin{array}{c} X_{final}=Conv\left(X_{cat}\right) \end{array}$$

### 3.4. Mamba_F

This module follows the design principles of the C3 module. Specifically, first, a 1 × 1 convolutional layer is used to integrate the feature channels. Next, the visual state space (VSS) blocks are used to encode the spatial features. The core of VMamba is a stack of visual state space (VSS) blocks with 2D Selective Scanning (SS2D) modules. Convolutional layers are also used to enhance local perception. Finally, the outputs of the VSS and convolutional layers are concatenated along the channel dimension, and 1 × 1 convolution is used to integrate the feature channels. Mamba_F can be represented as follows:

(10) $$\begin{array}{c} X_{in}=Conv\left(X\right)\left(\in R^{eC\times H\times W}\right) \end{array}$$

(11) $$\begin{array}{c} X_{mb}=VSS\left(X_{in}\right) \end{array}$$

(12) $$\begin{array}{c} X_{cv}=Conv\left(X_{in}\right) \end{array}$$

(13) $$\begin{array}{c} X_{cat}=Concat\left(X_{mb},X_{cv}\right)\left(\in R^{C\times H\times w}\right) \end{array}$$

(14) $$\begin{array}{c} X_{out}=Conv\left(X_{cat}\right) \end{array}$$

### 3.5. Feature Fusion

**Figure 5:**
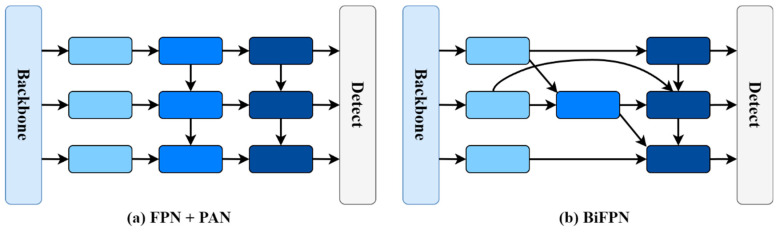
Bidirectional feature pyramid network structure.

In previous studies [48,49], some researchers have chosen to combine the Feature Pyramid Network (FPN) and the Path Aggregation Network (PAN) to fuse shallow and deep features. While this method can effectively fuse features, it requires converting all feature maps to the same resolution. Since the input feature contributions at different resolutions to the output features are unequal, this method cannot fully utilize feature information at different scales. Therefore, a bidirectional feature pyramid network [50] is used to fuse shallow and deep features, as illustrated in Figure 5. The bidirectional feature pyramid network enhances efficient and dynamic feature representation by fusing multi-scale features in a bidirectional, weighted, and repeated manner. Compared to the method combining the FPN and the PAN, it has three key improvements: removing nodes with only single-input edges, adding extra connections between the original input and output nodes at the same level, and reusing each bidirectional path as a feature network layer. These improvements enable the bidirectional pyramid structure to aggregate features of different scales and levels, thereby achieving more effective information integration. Overall, the bidirectional feature pyramid network not only enhances the integration capability of multi-scale features, but also significantly improves adaptability and detection accuracy in real traffic scenarios through repeated feature fusion and dynamic weighting mechanisms. At the same time, this design maintains high efficiency with regard to computational overhead.

## 4. Results

### 4.1. Dataset

This paper uses the UA-DETRAC dataset [51,52,53] to validate the effectiveness of the proposed algorithm. This dataset is a large open-source resource specifically designed for vehicle detection and tracking. The UA-DETRAC dataset records real traffic conditions from 24 roads in Beijing and Tianjin, covering four different weather conditions: sunny, rainy, cloudy, and night-time. The dataset classifies vehicles into categories like cars, buses, vans, and others, with a total of 8250 vehicles and 1.21 million labels.

**Figure 6:**
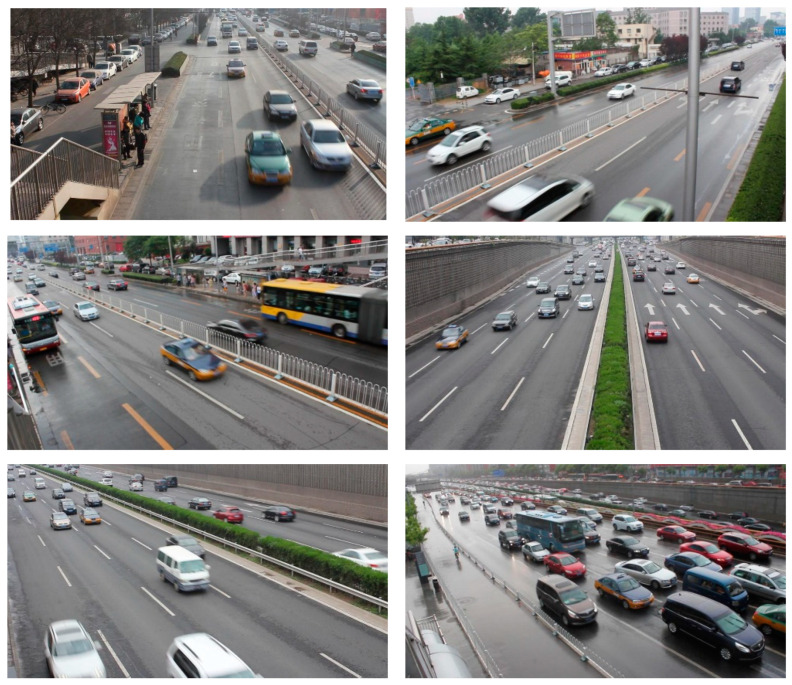
Sample images from the UA-DETRAC dataset.

The UA-DETRAC dataset consists of multiple videos, resulting in minimal variation in vehicles between adjacent video frames. Using the dataset directly for model training can lead to data redundancy. To address this, we extract frames from the original video at 10-frame intervals. This approach reduces the dataset size and model training time while avoiding overfitting due to repetitive feature learning. We selected 8639 images from the original UA-DETRAC training set as the new training set and 2231 images from the original UA-DETRAC test set as the new test set. Some of the training samples are shown in Figure 6.

Additionally, we chose the BDD100K dataset to verify the generalization ability and robustness of our method. BDD100K is a challenging dataset that covers almost all real traffic scenarios, including different times of the day, uncommon scenarios, and different weather conditions. A total of 20,955 images were randomly selected. The training set (16,764 images), validation set (2095 images), and test set (2096 images) were divided in an 8:1:1 ratio.

### 4.2. Experimental Equipment and Evaluation Metrics

The experiment was conducted on an Ubuntu 20.04 LTS operating system using hardware including an Intel Xeon Gold 6330 CPU (128 GB memory) and an RTX 3090 GPU (24 GB VRAM), Intel: Santa Clara, CA, USA. The Pytorch framework was employed for algorithm training, with CUDA version 11.8 and Pytorch version 2.1.1. The initial weight was set to 0.001, and the model was trained for a total of 100 epochs.

To evaluate the performance of the proposed algorithm, we used the mean Average Precision (mAP@50) and the number of model parameters as the primary evaluation metrics.

The formula for mAP@50 for nnn categories is as follows:

(15) $$\begin{array}{c} mAP@50=\frac{1}{n}\sum_{i=1}^{n}\int_{0}^{1}P\left(R\right)dR \end{array}$$

where P and R represent accuracy and recall, respectively.

(16) $$\begin{array}{c} P=\frac{TP}{TP+FP} \end{array}$$

(17) $$\begin{array}{c} R=\frac{TP}{TP+FN} \end{array}$$

where TP represents the number of correctly identified positive samples, FP represents the number of misidentified negative samples, and FN represents the number of missed positive samples.

### 4.3. Comparison Experiment

Currently, there is a large number of deep learning-based vehicle detection algorithms. To evaluate the performance of our proposed algorithm, we compared Mamba_ViT_YOLO with several existing classical vehicle detection algorithms on the UA-DETRAC dataset. The comparison algorithms include two-stage methods (Faster R-CNN) and single-stage methods (SSD, YOLOv3-tiny, YOLOv4-tiny, YOLOv5s, YOLOv6n, YOLOv7-tiny, YOLOv8-tiny). The specific results are shown in Table 1. As illustrated in Table 1, compared to the two-stage algorithm Faster R-CNN, Mamba_ViT_YOLO has only 4% of Faster R-CNN’s parameters, but its mAP@50 is 8% higher. The single-stage algorithm SSD has 13 times more parameters but 5.6% lower accuracy compared to Mamba_ViT_YOLO. Mamba_ViT_YOLO achieves a 9%, 8%, 6.4%, 5.2%, and 11.6% higher mAP@50 than YOLOv3-tiny, YOLOv4-tiny, YOLOv5s, YOLOv6n, and YOLOv7-tiny, respectively, while having significantly fewer parameters than these algorithms. Compared to the latest YOLOv8 algorithm, Mamba_ViT_YOLO has a 3.2% higher mAP@50 and only three-fifths of YOLOv8′s parameters. In summary, our approach performs well in real traffic scenarios and is able to remain lightweight. Notably, the computational complexity (Flops) of Mamba_ViT_YOLO is less than that of the other algorithms. The number of parameters and higher FLOPS of a deep learning model directly affect the model’s computational resource requirements and actual performance. Larger parameter counts and FLOPS tend to increase the computational overhead of the model, especially in resource-constrained environments. Our proposed algorithm achieves higher accuracy with a lower number of parameters and computational complexity. This demonstrates the suitability of our method for use on devices with limited computational resources. The specific reasons are as follows:

- Mamba_ViT utilizes two modules, Mamba_F and iRMB_F, to achieve effective separation and independent processing of shallow and deep features. By fully leveraging the strengths of both Mamba and the Transformer, it ensures comprehensive and accurate feature extraction. Its efficient feature extraction capability enables the network to better handle complex traffic scenarios and reduces detection errors caused by insufficient local features.
- The integration of the bidirectional pyramid feature fusion network into Mamba_ViT ensures thorough fusion of shallow and deep features. The bidirectional pyramid structure maximizes the complementarity between features at different layers, allowing shallow features to contribute detailed local information to deep features, while deep features provide global contextual information to shallow features, thereby enhancing the overall feature representation.
- The 2D selective scanning (SS2D) module in Mamba_F and the windowed attention in iRMB_F can effectively reduce the secondary computational complexity of attention, which greatly reduces the computational cost. In the feature fusion part, we use a bidirectional pyramid feature fusion network to reduce the model parameters while fully integrating the information. Compared to some advanced vehicle detection algorithms, higher accuracy is achieved with fewer parameters and computational complexity. Therefore, in theory, Mamba_ViT_YOLO fulfills the criteria of a lightweight algorithm.

In order to verify the generalizability and robustness of our method, we conducted relevant experiments on the BDD100K dataset. The specific experimental results are shown in Table 2. From Table 2, we can see that the accuracy of our algorithm is higher than all other algorithms. For example, compared with YOLOv3-tiny, YOLOv5n, and YOLOv7-tiny, the accuracy is improved by 14.4%, 2.8%, and 11.2%. As a result, the generalization and robustness of our method on the BDD100K dataset are further validated.

### 4.4. Ablation Experiment

In this section, we designed ablation experiments to validate the effectiveness of the proposed improvements to the algorithm, as shown in Table 3. Although Mamba_ViT combines the advantages of both the Transformer and Mamba, it does not cause a significant increase in the number of model parameters while enhancing the model’s ability to perceive vehicle information. The BiFPN (bidirectional feature pyramid network) effectively integrates shallow and deep features while reducing model parameters. Therefore, our proposed method successfully reduces the model parameters and improves the detection performance of the model in real-world scenarios.

### 4.5. Comparison of Heat Maps

**Figure 7:**
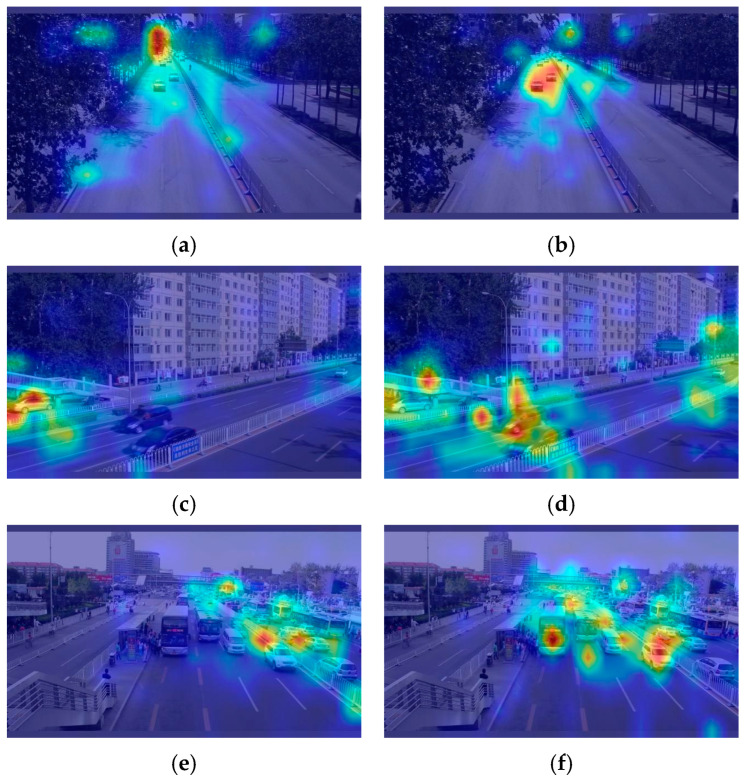
Heat map comparison results: (**a**,**c**,**e**) for YOLO8 heat map (**b**,**d**,**f**) for Mamba_ViT heat map.

A heat map is a visualization technique commonly used in target detection to show the intensity distribution of objects identified by the model in the input image. A heat map visualizes the location and confidence of the detection targets, where brighter regions indicate that the model has a higher confidence in the detection results. In Figure 7, we compare the heat maps generated by the YOLOv8 backbone network and Mamba_ViT.

Figure 7a,c,e show the heat maps generated by the YOLOv8 backbone network, showing that the level of focusing on the areas where vehicle targets are present is low and does not cover all areas where vehicles are present. Figure 7b,d,f show the heat maps generated by Mamba_ViT, where the darker areas are concentrated in the areas where vehicle targets are present and cover almost all of the areas where vehicle targets are present. In addition, we observe Mamba_ViT’s heat map and find that it is highly focused on regions where vehicle targets are present. These findings demonstrate that Mamba_ViT can comprehensively extract vehicle image features and make up for the local limitations of CNNs, enabling the model to accurately understand vehicle image information.

### 4.6. Comparison of Detection Results

**Figure 8:**
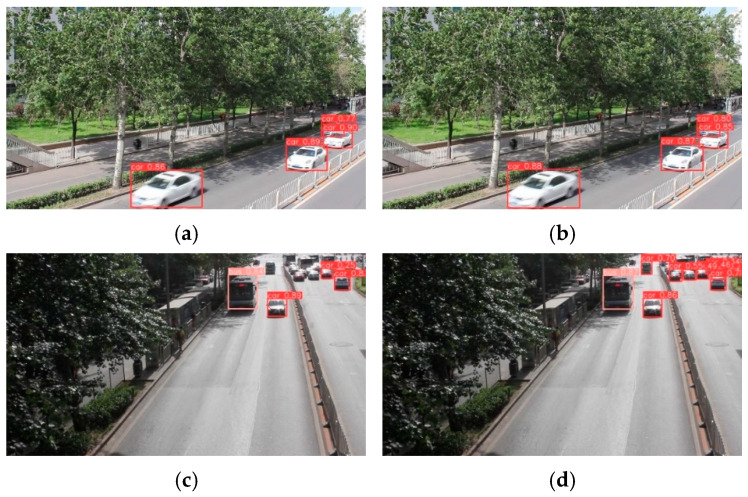

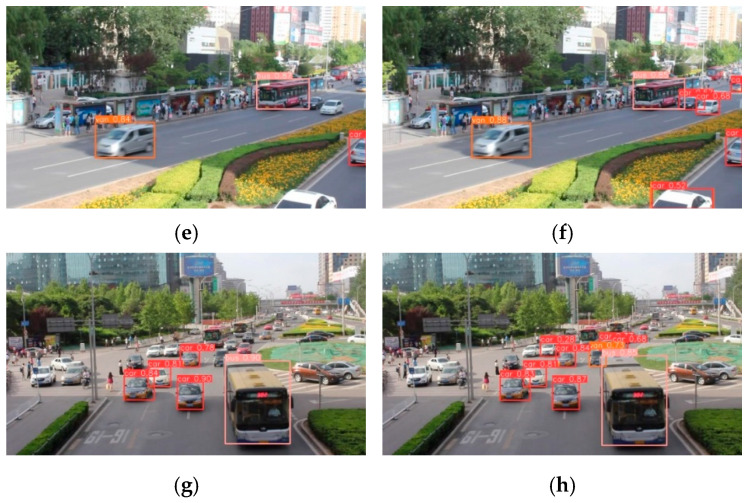
Comparison of detection results: (**a**,**c**,**e**,**g**) for YOLO8 detection results, (**b**,**d**,**f**,**h**) for Mamba_ViT_YOLO detection results.

We compare the images detected by YOLOv8 with our proposed model (Mamba_ViT_YOLO), as shown in Figure 8. Figure 8a,c,e,g show the images detected by YOLOv8, revealing that the YOLOv8 algorithm fails to detect all of the vehicle targets and has low detection accuracy. Figure 8b,d,f,h show the images detected by Mamba_ViT_YOLO, revealing that Mamba_ViT_YOLO is able to detect multiple vehicle targets efficiently and with good detection accuracy. This discrepancy may be due to the high vehicle traffic and vehicles occluding each other, resulting in missed detections. However, this limitation is overcome by utilizing Mamba_ViT and a bidirectional feature pyramid. Figure 8 also contains four different traffic scenarios. Our proposed algorithm detects vehicles better than the YOLOv8 algorithm in different scenarios. Therefore, our proposed algorithm is effective in detecting vehicles in real traffic scenarios and demonstrates high adaptability across different scenarios.

## 5. Discussion

Mamba_ViT_YOLO excels in feature extraction and multi-scale feature fusion and achieves high detection accuracy with fewer parameters and lower computational complexity. Despite these advantages, our approach still faces some challenges in practical applications. Firstly, although our method meets the standard of real-time detection in the speed method, it has not yet reached the ideal level. Second, the low computational complexity and small number of parameters of our method suggest that the method is well suited for devices with limited computational resources. However, due to the limitations of the experimental equipment, we have not yet tested the effectiveness of the method on mobile devices. Finally, the design concept of Mamba_ViT_YOLO is not limited to vehicle detection. Its feature extraction and multi-scale feature fusion capabilities make it potentially valuable for other object detection tasks, especially when handling diverse targets and complex scenarios. However, further research and validation are needed in this regard. Therefore, the focus will be on how the algorithm can be optimized further to accommodate the computational resource constraints of mobile devices, particularly in terms of inference speed, energy management, and model compression, and to validate its performance in various application scenarios, such as pedestrian detection and object classification. In addition to this, in the future, we plan to conduct relevant experiments in this area once equipment conditions allow for this.

## 6. Conclusions

To address the limitations of current vehicle detection algorithms in terms of insufficient feature extraction and large parameter counts, this paper proposes a lightweight vehicle detection model based on Mamba_ViT. We designed an efficient feature extraction network, Mamba_ViT, and the multi-scale feature fusion structure is incorporated into Mamba_ViT and used to construct the vehicle detection model Mamba_ViT_YOLO.

The experimental results show that the improved algorithm increases the average accuracy by 3.2% on the UA-DETRAC dataset compared to YOLOv8-tiny, and the number of parameters is only 0.6 times of that of YOLOv8-tiny. It outperforms SSD by 5.6% in terms of mAP@50. Compared to Faster-RCNN, this algorithm’s mAP@50 exceeds that of the other algorithm by 8%. Compared with YOLOv3-tiny, YOLOv4-tiny, YOLOv5s, YOLOv6n, and YOLOv7-tiny, this algorithm’s mAP@50 is 9%, 8%, 6.4%, 5.2%, and 11.6% higher, respectively, and the number of parameters is much smaller compared to these algorithms. The Mamba_ViT_YOLO algorithm shows excellent performance in different scenarios. The Mamba_ViT_YOLO algorithm achieves higher detection accuracy with fewer parameters and reduces dependency on storage and computational resources. Therefore, the algorithm is suitable for use on mobile devices with limited computational resources. In future research, we plan to further explore how the Mamba_ViT model can be deployed to embedded and edge devices, taking into account the computational resources and energy constraints of these environments. A key challenge is how to optimize the model to ensure real-time performance without sacrificing accuracy. In addition, maintaining the robustness of the model in various traffic scenarios, especially in low visibility or occlusion situations, is also a focus of our future research. We will also aim to reduce the memory footprint of the model to better suit resource-constrained device environments.

## Figures and Tables

**Table 1:** Comparison with other detection algorithms.

Model	Params (M)	FLOPS (G)	mAP@50
Faster-RCNN	41.3	60.5	0.508
SSD	24.1	30.5	0.532
YOLOv3-tiny	8.7	12.9	0.498
YOLOv4-tiny	6.0	16.2	0.508
YOLOv5s	7.0	16.0	0.524
YOLOv6n	4.6	11.3	0.536
YOLOv7-tiny	6.0	13.0	0.472
YOLOv8-tiny	3.0	8.1	0.556
Mamba_ViT_YOLO	1.8	6.1	0.588

**Table 2:** Comparison with other detection algorithms.

Model	Params (M)	FLOPS (G)	mAP@50	mAP@50:95
YOLOv3-tiny	8.7	12.9	0.254	0.11
YOLOv5n	1.8	4.2	0.37	0185
YOLOv7-tiny	6.0	13.0	0.286	0.153
Mamba_ViT_YOLO	1.8	4.2	0.398	0.22

**Table 3:** Ablation experiment.

YOLOv8	Mamba_ViT	BiFPN	Params (M)	mAP@50
√	×	×	3.0	0.556
√	√	×	2.8	0.575
√	×	√	2.0	0.57
√	√	√	1.8	0.588

## Data Availability

The original contributions presented in the study are included in the article, further inquiries can be directed to the corresponding author.

## References

[1] Wang Z., Zhan J., Duan C., Guan X., Lu P., Yang K. (2022). A review of vehicle detection techniques for intelligent vehicles. IEEE Trans. Neural Netw. Learn. Syst..

[2] Nigam N., Singh D.P., Choudhary J.J.S. (2023). A review of different components of the intelligent traffic management system (ITMS). Symmetry.

[3] Badi I., Bouraima M.B., Muhammad L.J. (2023). The role of intelligent transportation systems in solving traffic problems and reducing environmental negative impact of urban transport. Decis. Mak. Anal..

[4] Zhang Y., Sun Y., Wang Z., Jiang Y. (2023). YOLOv7-RAR for urban vehicle detection. Sensors.

[5] Bie M., Liu Y., Li G., Hong J., Li J. (2023). Real-time vehicle detection algorithm based on a lightweight You-Only-Look-Once (YOLOv5n-L) approach. Expert Syst. Appl..

[6] Bochkovskiy A., Wang C.Y., Liao H.Y.M. (2020). YOLOv4: Optimal Speed and Accuracy of Object Detection. arXiv.

[7] Wang C.-Y., Bochkovskiy A., Liao H.-Y.M. YOLOv7: Trainable bag-of-freebies sets new state-of-the-art for real-time object detectors. Proceedings of the IEEE/CVF Conference on Computer Vision and Pattern Recognition.

[8] Liu W., Anguelov D., Erhan D., Szegedy C., Reed S., Fu C.-Y., Berg A.C. Ssd: Single shot multibox detector. Proceedings of the Computer Vision–ECCV 2016: 14th European Conference.

[9] Yang Z., Yuan Y., Zhang M., Zhao X., Tian B. (2019). Safety Distance Identification for Crane Drivers Based on Mask R-CNN. Sensors.

[10] Li Z., Li Y., Yang Y., Guo R., Yang J., Yue J., Wang Y. (2021). A high-precision detection method of hydroponic lettuce seedlings status based on improved Faster RCNN. Comput. Electron. Agric..

[11] Wang C.C., Samani H., Yang C.Y. Object Detection with Deep Learning for Underwater Environment. Proceedings of the 2019 4th International Conference on Information Technology Research (ICITR).

[12] Yu W., Liu Z., Zhuang Z., Liu Y., Wang X., Yang Y., Gou B. (2022). Super-Resolution Reconstruction of Speckle Images of Engineered Bamboo Based on an Attention-Dense Residual Network. Sensors.

[13] Wang K., Liu M., Ye Z. (2021). An advanced YOLOv3 method for small-scale road object detection. Appl. Soft Comput..

[14] Kasper-Eulaers M., Hahn N., Berger S., Sebulonsen T., Myrland Ø., Kummervold P.E. (2021). Short Communication: Detecting heavy goods vehicles in rest areas in winter conditions using YOLOv5. Algorithms.

[15] Dong X., Yan S., Duan C. (2022). lightweight vehicles detection network model based on YOLOv5. Eng. Appl. Artif. Intell..

[16] Zhang X., Zhang X., He M. (2024). Research on vehicle detection method based on improved YOLOX-s. J. Syst. Simul..

[17] Elhanashi A., Saponara S., Dini P., Zheng Q., Morita D., Raytchev B. (2023). An integrated and real-time social distancing, mask detection, and facial temperature video measurement system for pandemic monitoring. J. Real-Time Image Process..

[18] Babenko A., Lempitsky V. (2015). Aggregating deep convolutional features for image retrieval. arXiv.

[19] Zhang Y., Zhao H., Duan Z., Huang L., Deng J., Zhang Q. (2021). Congested crowd counting via adaptive multi-scale context learning. Sensors.

[20] Zhu X., Lyu S., Wang X., Zhao Q. TPH-YOLOv5: Improved YOLOv5 based on transformer prediction head for object detection on drone-captured scenarios. Proceedings of the IEEE/CVF International Conference on Computer Vision.

[21] Sun Y., Wang W., Zhang Q., Ni H., Zhang X. Improved YOLOv5 with transformer for large scene military vehicle detection on SAR image. Proceedings of the 2022 7th International Conference on Image, Vision and Computing (ICIVC).

[22] Liu P., Fu H., Ma H. (2021). An end-to-end convolutional network for joint detecting and denoising adversarial perturbations in vehicle classification. Comput. Vis. Media.

[23] Lee D.-S. (2005). Effective Gaussian mixture learning for video background subtraction. IEEE Trans. Pattern Anal. Mach. Intell..

[24] Dalal N., Triggs B. Histograms of oriented gradients for human detection. Proceedings of the 2005 IEEE Computer Society Conference on Computer Vision and Pattern Recognition (CVPR’05).

[25] Viola P.A., Jones M.J. Rapid Object Detection using a Boosted Cascade of Simple Features. Proceedings of the 2001 IEEE Computer Society Conference on Computer Vision and Pattern Recognition. CVPR 2001.

[26] Lowe D.G. (2004). Distinctive Image Features from Scale-Invariant Keypoints. Int. J. Comput. Vis..

[27] Amit Y., Felzenszwalb P., Girshick R., Ikeuchi K. (2021). Object Detection. Computer Vision: A Reference Guide.

[28] Jheng Y.-J., Yen Y.-H., Sun T.-Y. A symmetry-based forward vehicle detection and collision warning system on Android smartphone. Proceedings of the 2015 IEEE International Conference on Consumer Electronics-Taiwan.

[29] Munajat M.E., Widyantoro D.H., Munir R. Vehicle detection and tracking based on corner and lines adjacent detection features. Proceedings of the 2016 2nd International Conference on Science in Information Technology (ICSITech).

[30] Satzoda R.K., Trivedi M.M. (2015). Multipart vehicle detection using symmetry-derived analysis and active learning. IEEE Trans. Intell. Transp. Syst..

[31] Zhang P.-p. (2010). Moving Target Detection and Tracking in Video Monitoring System. https://www.semanticscholar.org/paper/Moving-Target-Detection-and-Tracking-in-Video-Peng-pen/f46d58f1545bddcf49f0c5e339cf03c7f891d9b3.

[32] Wu X., Song X., Gao S., Chen C.J.T.M.T. Review of target detection algorithms based on deep learning. Proceedings of the CCEAI 2021: 5th International Conference on Control Engineering and Artificial Intelligence.

[33] Xie W., Zhu D., Tong X. (2013). Small target detection method based on visual attention. Jisuanji Gongcheng Yu Yingyong (Comput. Eng. Appl.).

[34] Yin S., Li H., Teng L. (2020). Imaging. Airport Detection Based on Improved Faster RCNN in Large Scale Remote Sensing Images. Sens. Imaging.

[35] Borji A., Cheng M.M., Jiang H., Li J. (2015). Salient Object Detection: A Benchmark. IEEE Trans. Image Process..

[36] Karangwa J., Liu J., Zeng Z. (2023). Vehicle detection for autonomous driving: A review of algorithms and datasets. IEEE Trans. Intell. Transp. Syst..

[37] Redmon J., Farhadi A. YOLO9000: Better, faster, stronger. Proceedings of the IEEE Conference on Computer Vision and Pattern Recognition.

[38] Jing L.I., Shan H. (2019). YOLOv3 Based Object Tracking Method. Electron. Opt. Control.

[39] Howard A., Sandler M., Chen B., Wang W., Chen L.C., Tan M., Chu G., Vasudevan V., Zhu Y., Pang R. Searching for MobileNetV3. Proceedings of the 2019 IEEE/CVF International Conference on Computer Vision (ICCV).

[40] Tan M., Le Q. (2019). EfficientNet: Rethinking Model Scaling for Convolutional Neural Networks. Proc. Mach. Learn. Res..

[41] Chen J., Kao S.-h., He H., Zhuo W., Wen S., Lee C.-H., Chan S.-H.G. Run, don’t walk: Chasing higher FLOPS for faster neural networks. Proceedings of the IEEE/CVF Conference on Computer Vision and Pattern Recognition.

[42] Liu Z., Hao Z., Han K., Tang Y., Wang Y. (2024). GhostNetV3: Exploring the Training Strategies for Compact Models. arXiv.

[43] He K., Zhang X., Ren S., Sun J. Deep Residual Learning for Image Recognition. Proceedings of the 2016 IEEE Conference on Computer Vision and Pattern Recognition (CVPR).

[44] Liu Z., Lin Y., Cao Y., Hu H., Wei Y., Zhang Z., Lin S., Guo B. Swin Transformer: Hierarchical Vision Transformer using Shifted Windows. Proceedings of the 2021 IEEE/CVF International Conference on Computer Vision (ICCV).

[45] Wang W., Xie E., Li X., Fan D.P., Shao L. Pyramid Vision Transformer: A Versatile Backbone for Dense Prediction without Convolutions. Proceedings of the 2021 IEEE/CVF International Conference on Computer Vision (ICCV).

[46] Zhang J., Li X., Li J., Liu L., Xue Z., Zhang B., Jiang Z., Huang T., Wang Y., Wang C. Rethinking mobile block for efficient attention-based models. Proceedings of the 2023 IEEE/CVF International Conference on Computer Vision (ICCV).

[47] Liu Y., Tian Y., Zhao Y., Yu H., Xie L., Wang Y., Ye Q., Liu Y.V. (2024). Mamba: Visual State Space Model. arXiv.

[48] Zheng Y., Zhang X., Zhang R., Wang D. (2022). Gated Path Aggregation Feature Pyramid Network for Object Detection in Remote Sensing Images. Remote Sens..

[49] Yu H., Li X., Feng Y., Han S.J.A.i. (2023). Multiple attentional path aggregation network for marine object detection. Appl. Intell..

[50] Tan M., Pang R., Le Q.V. Efficientdet: Scalable and efficient object detection. Proceedings of the IEEE/CVF Conference on Computer Vision and Pattern Recognition.

[51] Lyu S., Chang M.-C., Du D., Li W., Wei Y., Coco M.D., Carcagnì P., Schumann A., Munjal B., Dang D.-Q.-T. UA-DETRAC 2018: Report of AVSS2018 & IWT4S Challenge on Advanced Traffic Monitoring. Proceedings of the 2018 15th IEEE International Conference on Advanced Video and Signal Based Surveillance (AVSS).

[52] Lyu S., Chang M.-C., Du D., Wen L., Qi H., Li Y., Wei Y., Ke L., Hu T., Del Coco M. UA-DETRAC 2017: Report of AVSS2017 & IWT4S challenge on advanced traffic monitoring. Proceedings of the 2017 14th IEEE International Conference on Advanced Video and Signal Based Surveillance (AVSS).

[53] Wen L., Du D., Cai Z., Lei Z., Chang M.-C., Qi H., Lim J., Yang M.-H., Lyu S.J.C.V., Understanding I. (2020). UA-DETRAC: A new benchmark and protocol for multi-object detection and tracking. Comput. Vis. Image Underst..

